# Multicentric Angiographic Assessment of the Branching Patterns and Anastomotic Network of the Genicular Arteries, with Implications for Genicular Artery Embolization

**DOI:** 10.1007/s00270-025-04106-7

**Published:** 2025-07-10

**Authors:** Arian Taheri Amin, L. M. Wilms, N. Steinfort, D. Weiss, K. Jannusch, P. Freyhardt, M. Leist, C. Nolte-Ernsting, M. Katoh, A. Bücker, F. Ziayee, P. Minko

**Affiliations:** 1 Department of Diagnostic and Interventional Radiology, Medical Faculty, Dusseldorf University, 40225 Dusseldorf, Germany; 2 Institute for Diagnostic and Interventional Radiology, HELIOS Hospital Krefeld, Krefeld, Germany; 3 Clinic of Diagnostic and Interventional Radiology, Saarland University Hospital, Kirrberger Strasse Geb. 50.1, 66424 Homburg, Germany; 4 Department of Radiology, Evangelical Hospital Muelheim, Muelheim/Ruhr, Germany

**Keywords:** Genicular artery embolization, Genicular artery anatomy, Genicular artery anastomoses, Embolization technique, Nontarget embolization

## Abstract

**Purpose:**

To describe the anatomy of the genicular arteries and their anastomoses based on intraprocedural digital subtraction angiography (DSA).

**Materials and Methods:**

This retrospective, multi-center study reviewed patients who underwent genicular artery embolization (GAE) between January 2019 and December 2023. DSA images were analyzed to assess the anatomy of the genicular arteries and their anastomoses.

**Results:**

A total of 393 GAEs in 358 patients with minimal to severe knee osteoarthrosis (OA; Kellgren/Lawrence Grade I–IV) were analysed. Anastomoses between genicular branches were observed in all patients. In the medial compartment, anastomoses were identified between the descending genicular artery (DGA) and superior medial genicular artery (SMGA) in 158 GAEs (40%), the DGA and inferior medial genicular artery (IMGA) in 132 GAEs (34%), and the SMGA and IMGA in 64 GAEs (16%). In the lateral compartment, anastomoses were observed between the superior lateral genicular artery (SLGA) and inferior lateral genicular artery (ILGA) in 192 GAEs (49%), the ILGA and anterior tibial recurrent artery (ARTA) in 152 GAEs (39%), and between the SLGA, ILGA and ARTA in 91 GAEs (23%). Anastomoses between the medial and lateral compartments were identified between the DGA and SLGA in 59 GAEs (15%), the DGA and ILGA in 87 GAEs (22%), and the IMGA and ILGA in 94 GAEs (24%). The mean vessel diameter of the anastomoses ranged from 0.2 to 1.1 mm.

**Conclusion:**

Anastomoses between genicular arteries are frequent and extensive, with diameters exceeding the sizes of particles (100–300 μm) and microcatheters (1.7–2.4F) commonly used in GAE.

**Graphical Abstract:**

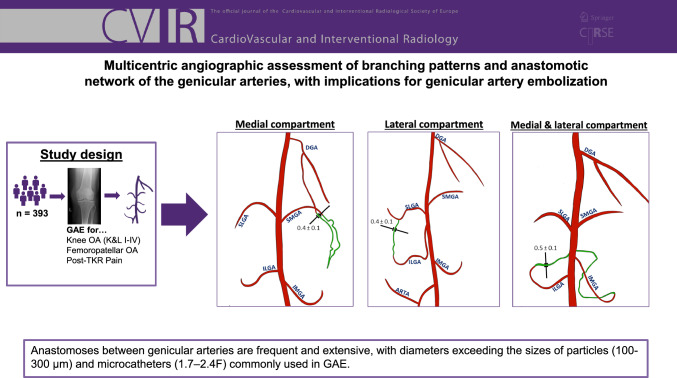

**Supplementary Information:**

The online version contains supplementary material available at 10.1007/s00270-025-04106-7.

## Introduction

Genicular artery embolization (GAE) is an emerging treatment for knee osteoarthritis (OA), recurrent hemarthrosis after total knee arthroplasty (TKA), and tendinopathy [1–4]. GAE targets abnormal neovascularization in the genicular arteries [5]. Meta-analyses have demonstrated its effectiveness and safety, showing improvements in pain, mobility, quality of life, and resolution of hemarthrosis post-TKA [6–9]. A detailed understanding of the genicular arteries–including anatomy, branching patterns, and anastomoses–is crucial for achieving technical success and minimizing complications. In addition to supplying the knee joint, these arteries also provide blood flow to the surrounding skin, muscles, tendons, ligaments, and bone, making accurate targeting essential to avoid non-target embolization. A better understanding of the anatomy may help reduce adverse events like periarticular skin ischemia and rare complications such as bone infarction, fat necrosis, and temporary paresthesia [10–12].

The origin variants and branching patterns of the genicular arteries have been extensively studied using cadaveric studies and cone-beam CT (CBCT) imaging [13–19]. However, angiographic analyses to date have been limited to small cohorts [19, 20], and detailed assessment of the branching patterns of the genicular arteries beyond their origins have not yet been conducted.

The aim of this study was to translate the origin variants, branching patterns, and anastomotic networks of the genicular arteries onto two-dimensional angiograms as encountered by any interventional radiologist performing GAE.

## Material and Methods

This retrospective multi-center study was conducted at the University Hospital of Duesseldorf (Germany), Saarland University Hospital (Germany), Helios Hospital Krefeld (Germany), and the Evangelical Hospital Muelheim an der Ruhr (Germany). The study was approved by the Institutional Review Board of each participating institute and conducted in accordance with the Declaration of Helsinki.

Records of patients with knee OA who underwent GAE between January 2019 and December 2023 were accessed with prior approval from the relevant authorities. Inclusion criteria were patients aged 18–100 years, who underwent GAE for therapy refractory knee pain due to knee OA, isolated femoropatellar OA, or following total knee arthroplasty. Patients with non-OA-related conditions, including rheumatologic or inflammatory joint diseases, were screened and excluded to minimize confounding factors. Exclusion criteria included cases with poor image quality inadequate for anatomical assessment or patients with alternative diagnoses contributing to knee pain that could impact the vascular anatomy assessment.

GAE was performed by board-certified interventional radiologists according to previously described techniques [1, 2, 21]. Interventional radiologists’ experience was as follows: F.Z., and P.M. had 13–18 years of experience; P.F. and M.K. had 18–24 years; M.L. and A.B. had 8 and > 30 years; C.N.-E. had > 25 years of experience. Following transfemoral access, angiography was performed at the mid-third of the distal superficial femoral artery (SFA) using a 3.3–4 F catheter to visualize the complete anatomy of the genicular arteries between the origins of the descending genicular artery (DGA) and the anterior tibial recurrent artery (ARTA). Subsequently, superselective catheterization of the genicular arteries was performed using a 1.7–2.4 F microcatheter. Genicular artery branches were embolized, using either permanent (100–300 μm Embospheres, Merit Medical, USA) or temporary (Imipenem/Cilastatin) embolic agents. Embolic agent selection was not standardized across centers and was determined at the discretion of the interventional radiologist based on local protocols and patient-specific factors.

In all patients, radiographs of the knee were reviewed by board certified radiologists, and OA was graded using the Kellgren-Lawrence–Scale (K&L) as doubtful (K&L Grade I), mild (K&L Grade II), moderate (K&L Grade III), or severe (K&L Grade IV). A research associate (NS) and two radiologists (AT, with 3 years of experience, and LW, with 9 years of experience in diagnostic vascular imaging), reviewed all digital subtraction angiography (DSA) images in consensus. In ambiguous cases, the assessments were further validated by two senior interventional radiologists (FZ and PM), with 13–18 years of experience, respectively, whose evaluations were considered decisive. However, no corrections were necessary. The following vessels were analyzed: Descending genicular artery (DGA), superior medial genicular artery (SMGA), inferior medial genicular artery (IMGA), superior lateral genicular artery (SLGA), inferior lateral genicular artery (ILGA), medial genicular artery (MGA), and anterior recurrent tibial artery (ARTA). For each genicular artery, the diameter measured within 0.5 cm of its origin, the branching pattern and the anastomotic network were recorded. To assess anastomoses, genicular arteries were categorized into three compartments: the medial compartment (i.e., DGA, SMGA, and IMGA), the lateral compartment (i.e., SLGA, ILGA, and ARTA), and the cruciate compartment (i.e., MGA). For each anastomosis, the diameter was measured within 0.5 cm of its origin from the catheterized genicular artery, using non-calibrated DSA images.

Radiographs and DSA images were reviewed using the Sectra DICOM viewer (Sectra AB, Linköping, Sweden). Descriptive statistical analyses were performed using SPSS (version 29.0.2, IBM, Armonk, USA).

## Results

A total of 358 patients were included in the study (Table 1). In 35 patients, both knees were treated, resulting in a total of 393 GAEs analyzed. Two patients (two GAEs) were excluded because of poor image quality. GAEs were performed for knee OA (K&L Grade I–IV), isolated femoropatellar OA and post-TKA pain.

**Table 1:** Patient characteristics

	Total	University Hospital Duesseldorf	Saarland University Hospital	Helios Hospital Krefeld	Evangelical Hospital Muelheim
Number of patients	358	176	66	79	37
Age (years, median; [age range])	69 [38–98]	68 [41–92]	66 [38–91]	66 [41–93]	82 [44–98]
Female (n, [%])	195 (54)	103 (59)	33 (50)	53 (67)	24 (65)
Number of knees (n)	393	187	71	97	38
Knee (n, [%])					
Right	204 [52]	104 [56]	31 [44]	52 [54]	17 [45]
Left	189 [48]	83 [44]	40 [56]	45 [46]	21 [55]
Indication (n)					
Knee OA	318	166	49	76	27
Femoropatellar OA	33	9	5	13	6
Post-TKA pain	42	12	17	8	5
Kellgren–Lawrence grade (n)					
I	2	0	0	1	1
II	39	3	12	16	8
III	132	52	19	47	14
IV	173	120	19	24	10

*OA* osteoarthritis; *TKA* total knee arthroplasty

**Fig. 1:**
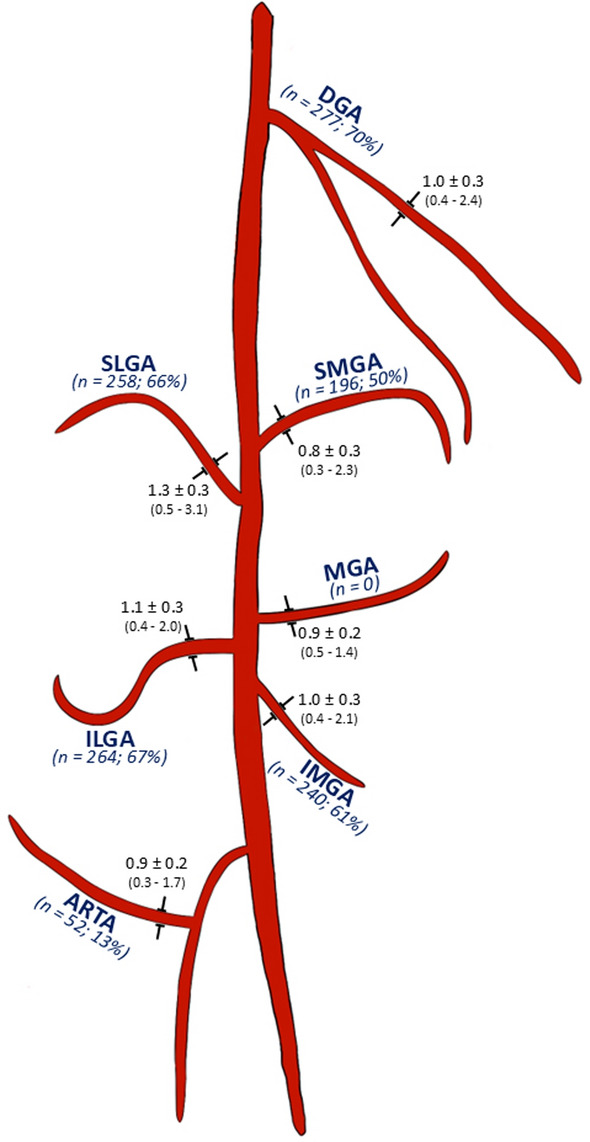
Diameters of genicular arteries. Schematic representation of genicular arteries and number of cases, in which each artery was embolized. Mean vessel diameter of the anastomoses in mm ± SD; *DGA* descending genicular artery; *SMGA* superior medial genicular artery; *IMGA* inferior medial genicular artery; *SLGA* superior lateral genicular artery; *ILGA* inferior lateral genicular artery; *MGA* Medial genicular artery; *ARTA* anterior recurrent tibial artery

A total of 1,287 genicular arteries were embolized (Fig. 1). Lateral DSA images were obtained in 156 GAEs (40%). Table 2 provides an overview of the prevalence and primary branching patterns of each genicular artery identified in our GAE cohort.

**Table 2:** Genicular artery occurrence and branching patterns in GAE

Genicular artery	Prevalence in GAEs (n, %)	Branching patterns (n, %)
Descending genicular artery (Fig. E1)	376 (96)	Articular branch (AB): 366 GAEs (93) Single AB: 178 GAEs (49) AB with medial & lateral branches: 188 GAEs (51)
Superior medial genicular artery (Fig. E2)	379 (96)	Single branch: 299 GAEs (79) Double branch: 54 GAEs (14) Additional cranial branches: 26 GAEs (7)
Inferior medial genicular artery (Fig. E3)	379 (96)	Single branch: 191 GAEs (50) Additional inferior branches: 153 GAEs (40) Additional crural branches: 33 GAEs (9)
Superior lateral genicular artery (Fig. E4)	391 (99)	Single branch: 13 GAEs (3) Additional superior branches: 378 GAEs (97)
Inferior lateral genicular artery (Fig. E5)	387 (98)	Single branch: 328 GAEs (85) Additional inferior branches: 59 GAEs (15)
Medial genicular artery (Fig. 1)	375 (95)	No additional branches: 375 GAEs (95)
Anterior recurrent tibial artery (Fig. 1)	387 (98)	No additional branches: 368 GAEs (95) Branching not evaluated in 19 GAEs (5) due to incomplete visualization

*GAE* genicular artery embolization

### Origin Variants

Independent origin and ramification of the DGA into the three common branches—muscular branch (MB), articular branch (AB) and saphenous branch (SB)–—was observed in 229 GAEs (61%). The AB was absent in 10 GAEs (3%), the MB in 12. GAEs (3%), and the SB in 22 GAEs (6%). In 8 GAEs (2%), the AB, and in 95 GAEs. (25%) the SB originated directly from the SFA (n = 74) or the P1 Segment (n = 21) of the popliteal artery.

A common trunk of the SLGA, SMGA, and the MGA was identified in 132 GAEs (35%) (see Fig. E6A). A common origin of the SLGA and MGA, with an independent origin of the SMGA was observed in 121 GAEs (32%) (see Fig. E6B). A common origin of the SMGA and MGA, with an independent origin of the SLGA was seen in. 13 GAEs (4%) (see Fig. E6C). Independent origins of the SMGA, SLGA, and MGA Were noted in 109 GAEs (28%).

A common origin of the ILGA and IMGA was seen in 10 GAEs (3%) and a common. origin of the IMGA and MGA in 6 GAEs (2%). Independent origin of the ILGA and. IMGA were identified in 363 GAEs (93%).

### Anastomoses

**Fig. 2:**
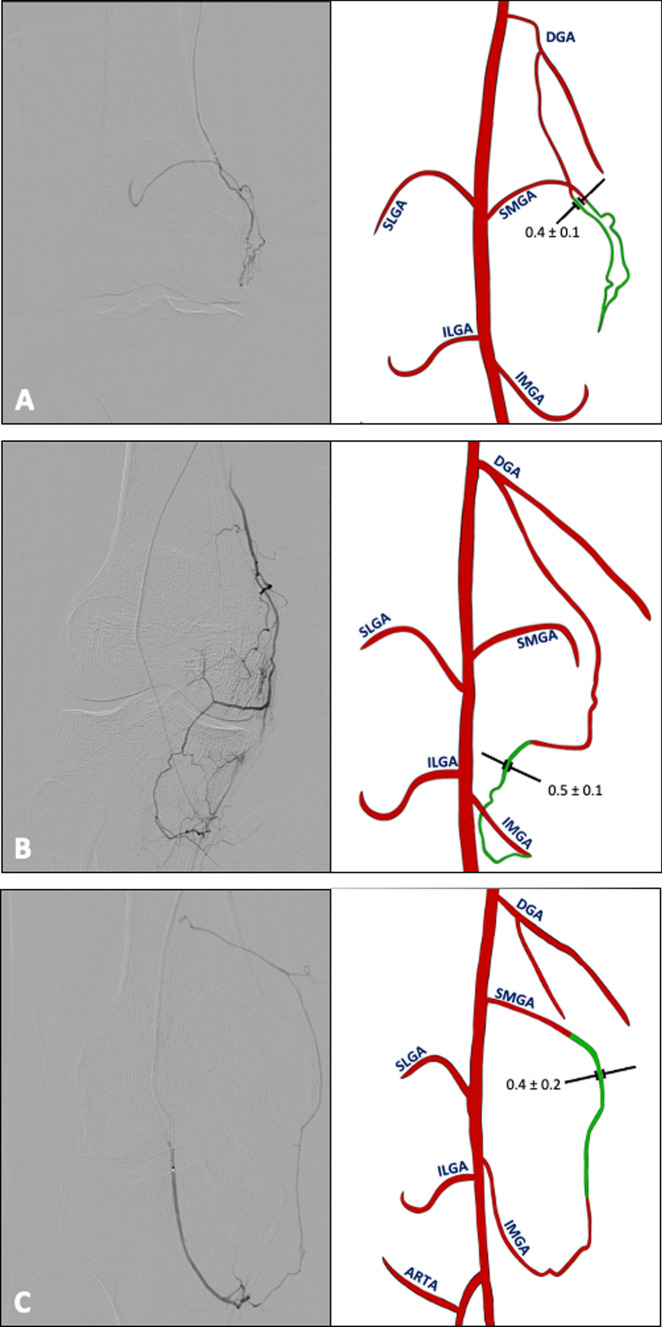
Anastomoses of the medial compartment of the knee Angiographic (left) and schematic (right) representations of anastomoses in the medial compartment in representative knee joints. Anastomoses (green) between DGA + SMGA **A** were seen in 158, between DGA + IMGA **B** in 133 and between SMGA + IMGA **C** in 65 GAEs. Mean diameter of the anastomoses in mm ± SD; *DGA* descending genicular artery; *SMGA* superior medial genicular artery;* IMGA* inferior medial genicular artery; *SLGA* superior lateral genicular artery*; ILGA* inferior lateral genicular artery; *ARTA* anterior recurrent tibial artery

**Fig. 3:**
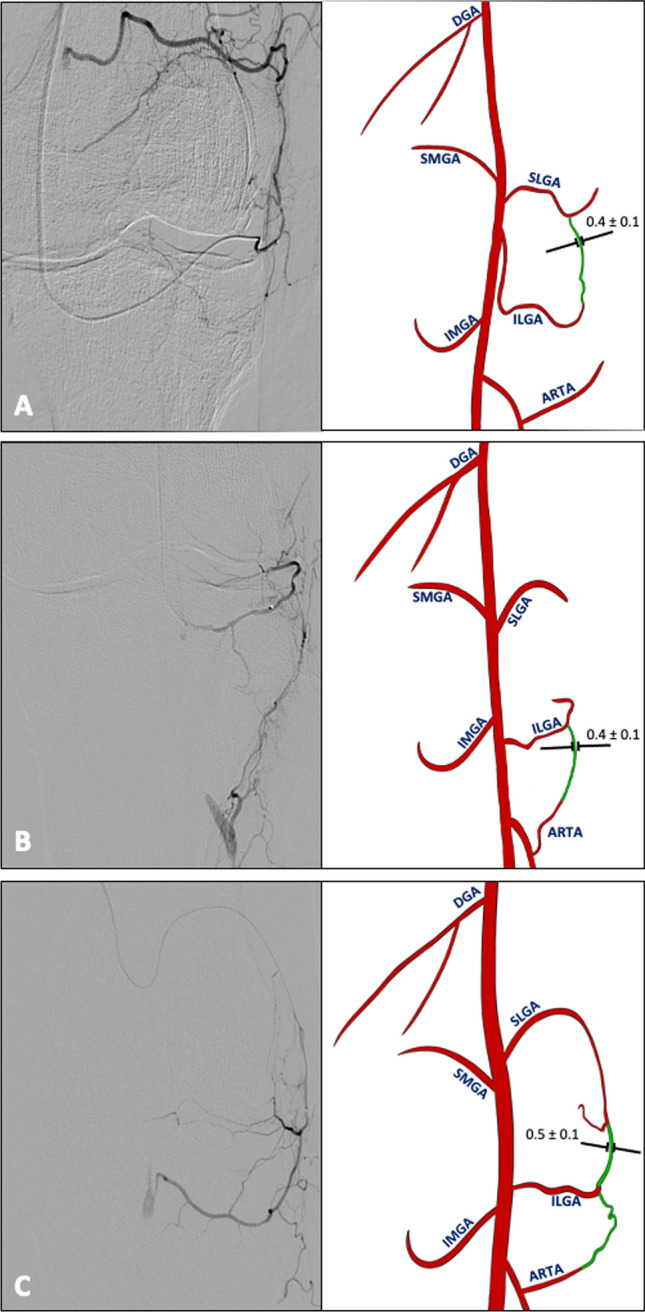
Anastomoses of the lateral compartment of the knee Angiographic (left) and schematic (right) representation of anastomoses in the lateral compartment in representative knee joints. Anastomoses (green) were observed between SLGA + ILGA **A** in 151 DSA images, **B** between ILGA + ARTA in 109 DSA images and between SLGA + ILGA + Arta **C** in 91 DSA images. Mean diameter of the anastomoses in mm ± SD; *DGA* descending genicular artery; *SMGA* superior medial genicular artery; *IMGA* inferior medial genicular artery; *SLGA* superior lateral genicular artery; *ILGA* inferior lateral genicular artery; *ARTA* anterior recurrent tibial artery

**Fig. 4:**
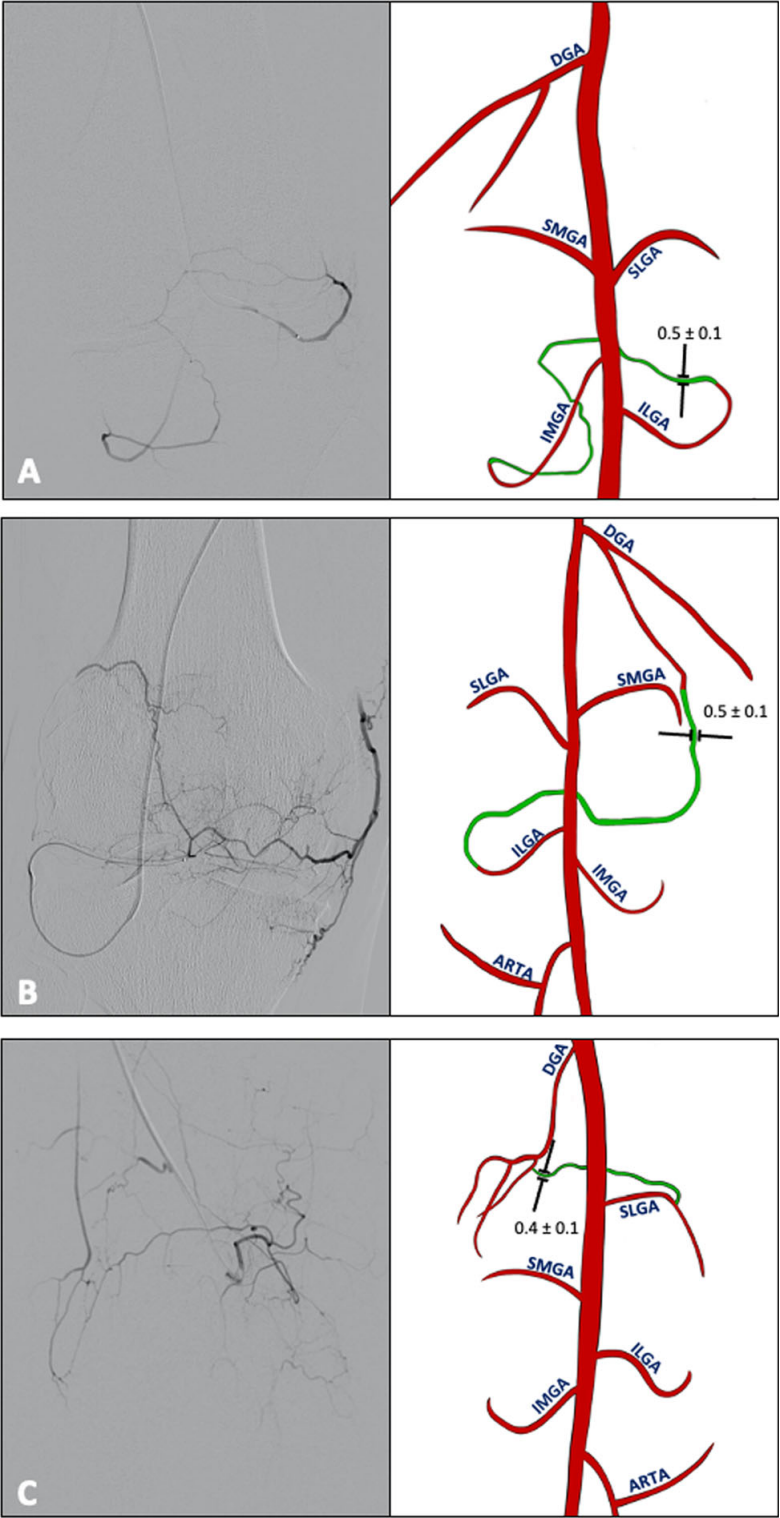
Anastomoses between the medial and lateral compartment of the knee Angiographic (left) and schematic (right) representation of anastomoses in the lateral compartment in representative knee joints. Anastomoses (green) were observed between IMGA + ILGA **A** in 97 DSA images, **B** between DGA + ILGA in 87 DSA images and between DGA + SLGA **C** in 59 DSA images. Mean diameter of the anastomoses in mm ± SD; *DGA* descending genicular artery; SMGA superior medial genicular artery; *IMGA* inferior medial genicular artery; *SLGA* superior lateral genicular artery; *ILGA* inferior lateral genicular artery; *ARTA* anterior recurrent tibial artery

Anastomoses were observed in all GAEs, with a total of 1077 anastomoses identified (Table 3). One anastomosis was observed in 109 GAEs (28%), two in 70 GAEs (18%), three in 60 GAEs (15%), four in 60 GAEs (15%), five in 32 GAEs (8%), six in 27 GAEs (7%), seven in 18 GAEs (5%), eight in 14 GAEs (4%) and nine in 3 GAEs (1%). Anastomoses were observed in the medial compartment (Fig. 2), the lateral compartment (Fig. 3), the medial and lateral compartment (Fig. 4), and the cruciate compartment.

**Table 3:** Anastomoses in knee compartments: frequency, caliber, and key patterns

Knee compartment	Total anastomoses (% of total)	Mean caliber (mm)	Key anastomosis patterns n (% of total)
Medial	356 (33)	0.5 ± 0.1 (0.2–1.0)	DGA and SMGA: 158 GAEs (15) DGA and IMGA: 133 GAEs (12) SMGA and IMGA: 65 GAEs (6)
Lateral	372 (35)	0.4 ± 0.1 (0.2–1.1)	SLGA and ILGA: 151 GAEs (14) ILGA and ARTA: 109 GAEs (10) SLGA, ILGA and ARTA: 91 GAEs (8) SLGA and ARTA: 21 GAEs (2)
Medial and lateral	346 (32)	0.4 ± 0.1 (0.2–1.0)	MGA and ILGA: 97 GAEs (9) DGA and ILGA: 87 GAEs (8) DGA and SLGA: 59 GAEs (5) IMGA and ARTA: 36 GAEs (3) SMGA and ILGA: 23 GAEs (2) SMGA and SLGA: 18 GAEs (2) SLGA and IMGA: 12 GAEs (1) DGA and ARTA: 10 GAEs (1) SMGA and ARTA: 4 GAEs (0.4)
Cruciate	3 (0.3)	0.1 ± 0.1 (0.2–0.5)	IMGA and MGA: 3 GAEs (0.3)

*GAE* genicular artery embolization; *DGA* descending genicular artery; *SMGA* superior medial genicular artery; *IMGA* inferior medial genicular artery; *SLGA* superior lateral genicular artery; *ILGA* inferior lateral genicular artery; *ARTA* anterior tibial recurrent artery; *MGA* middle genicular artery

## Discussion

This multicenter study provides a detailed angiographic analysis of genicular artery anatomy and its anastomotic network in a large cohort, emphasizing its complexity and variability. This should be carefully considered during GAE, as embolized vessels could be well-collateralized and remain perfused through anastomotic pathways.

Angiographic prevalence of origin variants partly differed from previous CBCT studies [13, 14]. Similar to Callese et al. [13], separate origins of genicular arteries were identified in 28%, while a common SMGA-MGA origin was identified in 4%. However, a common SLGA-MGA origin was less frequently in DSA (32%) than in CBCTs, whereas trifurcation of the SLGA, SMGA, and MGA occured nearly three times as often as reported previously [13]. Our larger sample size and differences between CBCT’s three-dimensional imaging and DSA’s projection-based visualization likely explain these discrepancies. As a radiation-sparing alternative, lateral DSA images were acquired in only 40% of cases to guide challenging catheterizations and prevent non-target embolization, especially of the SMGA.

The branching patterns of the genicular arteries have mainly been described in cadaveric studies [6, 15, 16, 19, 22–28]. In our study, 50% of the GAEs showed a bifurcation of the DGA’s articular branch. Medial and lateral branches, correspond to the longitudinal branch and transverse branch, described in cadaveric studies [22, 23]. Since the longitudinal branch supplies medial parts of the joint capsule [6, 22], superselective catheterization should be considered for tendinopathies.

A doubled SMGA was observed in 15% of GAEs. In a cadaveric study, a second, more distal branch of the SMGA was described occasionally [24]. Thus, the presence of a small-caliber SMGA should prompt angiographic assessment for a second vessel, and in cases of a doubled SMGA, catheterization of both branches may be warranted.

In nearly 50% of GAEs, an additional IMGA inferior branch was observed, extending toward the lower leg in 9% of GAEs. This branch, unreported in literature, correlates with previously noted anastomoses between the IMGA and the medial sural artery [19]. Since the medial sural artery supplies the medial gastrocnemius muscle and tibial nerve, the IMGA should be embolized distal to this anastomosis to prevent complications such as muscle necrosis or plantar paresthesia [10, 25, 26].

Almost all GAEs demonstrated bifurcation of the SLGA into and caudal branches corresponding to the deep articular and superficial patellar branch described in cadaveric studies [15, 19, 27]. In cases of isolated femoropatellar OA, superselective embolization of the superficial patellar branch may be advantageous.

In 15% of GAEs, additional inferior ILGA branches were observed, paralleling cadaveric studies [16, 19, 28]. For insertion tendinopathies or tibiofibular OA, superselective embolization of these branches can be considered.

The knee’s anastomotic network (“rete articulare genus”) is extensive, with a total of 1077 anastomoses identified; each patient exhibited at least one anastomosis of the genicular arteries:

In the medial compartment, anastomoses most frequently involved the DGA and SMGA [19, 20], followed by DGA-IMGA anastomoses, which have not yet been reported in angiographic studies [6, 19, 20]. A cadaveric study found that anastomoses between the IMGA and superficial patellar branch of the DGA were observed in all lower-extremity specimens [29]. Since the superficial patellar branch supplies musculocutaneous and neural structures of the superomedial knee [22, 30, 31], distal embolization is advised when targeting the IMGA. Less frequently, SMGA-IMGA anastomoses were observed, which remain unreported in angiographic studies.

In the lateral compartment**,** an anastomosis between SLGA and ILGA was most frequent, followed by an anastomosis between ILGA and ARTA, and an anastomosis involving the SLGA, ILGA, and ARTA. Cadaveric studies likewise identified SLGA-ILGA anastomosis as the most common, primarily supplying the lateral femoral condyle [24, 32, 33]. The anastomoses between the ILGA and ARTA, and the SLGA, ILGA, and ARTA, were also frequently described, though no prevalence rates were reported [32, 33]. Since these anastomoses allow embolic agents to reach the anterior tibial artery, they pose a risk of severe non-target embolization.

Cross-compartment anastomoses between the medial and lateral compartment (e.g., IMGA–ILGA, DGA–SLGA, DGA–ILGA) were also identified, aligning with other angiographic and cadaveric studies [20, 32–34]. Their higher prevalence compared to Bagla et al. [20] likely reflects interventional factors (i.e., number of catheterized branches, depth of catheterization) and our considerably larger multicenter study population.

With a mean anastomotic diameter of 0.4 mm—exceeding the size of standard 1.7 F microcatheters—retrograde catheterization through anastomoses may be feasible in complex or vasospastic cases if antegrade approaches prove difficult.

This study has several limitations. First, its retrospective multicenter design limits control over imaging protocols and intervention factors, since angiographic images were obtained during clinical routine. Differences in DSA settings, catheterization depth, contrast volumes, injection pressure and embolic materials may have affected vessel diameter measurements or led to omission of contrast-poor vessels. Exclusive reliance on DSA images precludes correlation with other imaging modalities, such as CBCT, which could offer complementary anatomical insights. This limitation was partially mitigated by acquiring lateral projections in 40% of challenging cases.

Second, while this study provides a detailed anatomical assessment, it does not include clinical outcomes (e.g., pain reduction or procedural efficacy), limiting direct clinical applicability. Furthermore, adverse events and complications were not systematically recorded**,** preventing analysis of complications after non-target embolization. The clinical significance of these anatomical variations remains to be determined through further studies that assess their correlation with procedural success and symptom relief.

Third, the inclusion of post-TKA patients may have introduced bias due to altered vascular anatomy, potentially making findings less representative of native genicular artery anatomy. However, these cases were included to reflect the real-world diversity of patients undergoing GAE. Future studies may benefit from analyzing post-TKA patients separately to better delineate anatomical differences.

Despite these limitations, the large cohort size and multicenter design provide a comprehensive overview of the genicular artery anastomotic network, offering valuable insights for procedural planning in genicular artery embolization (GAE). Future research correlating anatomical findings with clinical outcomes will be essential to refine embolization strategies, improve patient selection, and enhance procedural safety.

## Conclusion

The branching patterns and anastomotic network of the genicular arteries are complex and highly variable. The anatomy described in cadaveric studies, often derived from surgical works, does not fully address the anatomical considerations crucial for GAE. Comprehensive in-vivo anatomical knowledge—particularly as visualized in angiograms—combined with thorough pre-interventional assessment enables targeted treatment of the genicular artery branches based on each patient's clinical needs, while minimizing the risk of non-target embolization. Additionally, this knowledge helps identify alternative catheterization routes if antegrade access to a genicular branch proves challenging.

## Supplementary Information

Below is the link to the electronic supplementary material.

Supplementary file1 (DOCX 1119 KB)
