# Supplementary material for: Multicentric Angiographic Assessment of the Branching Patterns and Anastomotic Network of the Genicular Arteries, with Implications for Genicular Artery Embolization
Source: Cardiovasc Intervent Radiol. 2025 Jul 10;48(8):1142–51. doi: 10.1007/s00270-025-04106-7 (PMC12325466; doi:10.1007/s00270-025-04106-7)
Supplement: Supplementary file 1 — Supplementary file1 (DOCX 1119 KB) [file 270_2025_4106_MOESM1_ESM.docx]

**Figure E1: Common branches of the DGA**

The DGA (red) ramifies into 3 main branches: the muscular branch (blue), the articular branch (purple) and the saphenous branch (yellow). In 188 of GAEs (51%), the articular branch bifurcates into a transverse branch (green) and longitudinal branch (orange).


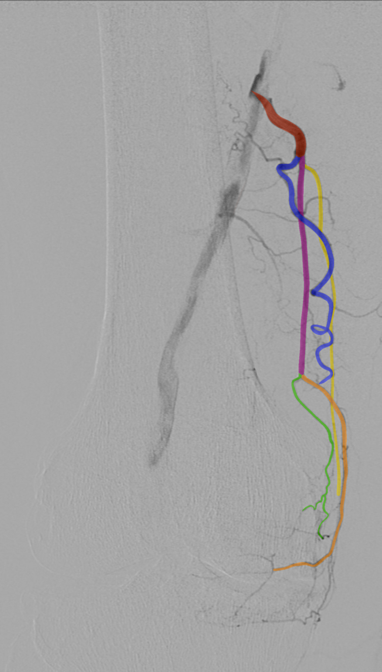


**Figure E2: Common branching pattern of the SMGA**

A doubled branch SMGA (red and blue) was observed in 54 of GAEs (14%), while ramification of the superior SMGA in superior (green) and inferior (orange) branches was seen in 24 of GAEs (7%).


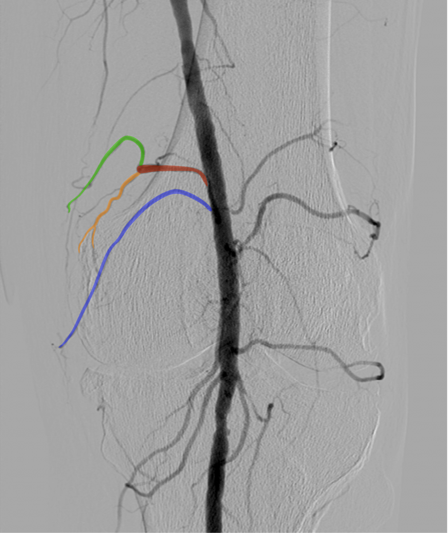


**Figure E3: Common branching pattern of the IMGA**

Caudal branches (orange) of the IMGA (red) descending toward the medial sural artery were seen in 191 of GAEs (50%).


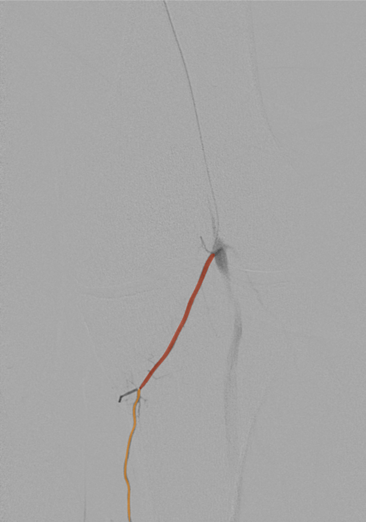


**Figure E4: Common branching pattern of the SLGA**

Bifurcation of the SLGA (red) into superior (orange) and inferior (green) branches was identified in 378 of GAEs (97%). Septocutaneous branches (purple) were frequently observed.


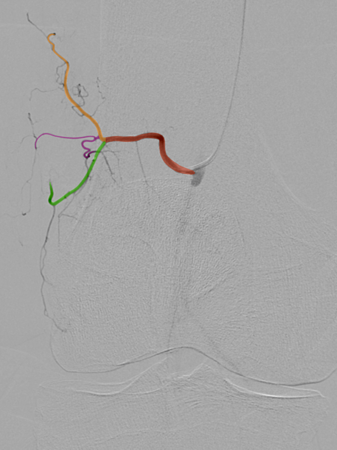


**Figure E5: Common branching pattern of the ILGA**

Additional inferior branches (yellow) of the ILGA (red) were observed in 59 of GAEs (15%).


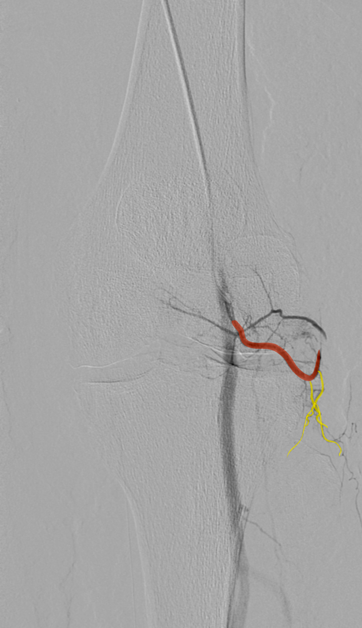


**Figure E6: Origin variants of the SMGA, SLGA and MGA.**

Trifurcation (A) of the SMGA (red), SLGA (green) and MGA (orange) was identified in 132 of GAEs (35%). A common origin of the SLGA and MGA (B) was observed in 121 (32%) and of the SMGA and MGA (C) in 13 of patients (4%).

**B**

**A**


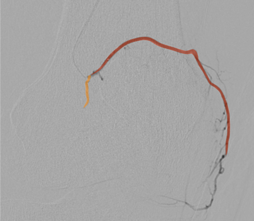


**C**


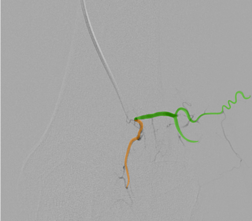

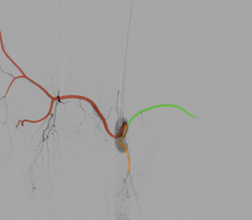


**B**

**C**
